# Arterial Aging and Cerebrovascular Function: Impact of Aerobic Exercise Training in Older Adults

**DOI:** 10.14336/AD.2023.1109-1

**Published:** 2024-08-01

**Authors:** Tsubasa Tomoto, Rong Zhang

**Affiliations:** ^1^Human Informatics and Interaction Research Institute, National Institute of Advanced Industrial Science and Technology (AIST), Tsukuba, Ibaraki, Japan.; ^2^Institute for Exercise and Environmental Medicine, Texas Health Presbyterian Hospital Dallas, Dallas, Texas, USA.; ^3^Departments of Neurology,; ^4^Internal Medicine, and; ^5^Biomedical Engineering, University of Texas Southwestern Medical Center, Dallas, Texas, USA

**Keywords:** age, aerobic exercise training, arterial stiffness, cerebral blood flow, cognitive function

## Abstract

Advanced age is the major risk factor for dementia including Alzheimer’s disease. The clinical effects of recently developed anti-amyloid therapy for Alzheimer’s disease were modest and the long-term outcome is unknown. Thus, an in-depth understanding of the mechanisms of brain aging is essential to develop preventive interventions to maintain cognitive health in late life. Mounting evidence suggests that arterial aging manifested as increases in central arterial stiffness is associated closely with cerebrovascular dysfunction and brain aging while improvement of cerebrovascular function with aerobic exercise training contributes to brain health in older adults. We summarized evidence in this brief review that 1) increases in central arterial stiffness and arterial pulsation with age are associated with increases in cerebrovascular resistance, reduction in cerebral blood flow, and cerebrovascular dysfunction, 2) aerobic exercise training improves cerebral blood flow by modifying arterial aging as indicated by reductions in cerebrovascular resistance, central arterial stiffness, arterial pulsation, and improvement in cerebrovascular function, and 3) improvement in cerebral blood flow and cerebrovascular function with aerobic exercise training may lead to improvement in cognitive function. These findings highlight the associations between arterial aging and cerebrovascular function and the importance of aerobic exercise in maintaining brain health in older adults.

## Introduction

The incidence of dementia continues to increase with the rapidly aging global population [1]. The major risk factor for dementia is advanced age [1, 2]. The clinical effects of recently developed anti-amyloid therapy for Alzheimer’s disease (AD), the most common type of dementia, were modest and the long-term outcome is unknown [3]. Therefore, an in-depth understanding of brain aging and its association with neurodegenerative diseases such as AD are essential to develop preventive interventions to preserve cognitive vitality or delay the onset or the progression of cognitive impairment associated with AD [1, 2, 4, 5]. In this regard, AD pathology can begin ~20 years before the onset of cognitive impairment [1, 4]. Accordingly, effective preventive interventions may need to start early in cognitively normal older adults or those with mild cognitive impairment (MCI: a prodromal stage of AD) [1, 5].

The transformation from healthy brain aging to AD is a complex process involving a combination of genetic, lifestyle, and environmental factors affecting the brain over a long period [1, 4, 6]. Accumulating evidence has demonstrated that the presence of cardiovascular diseases or its risk factors increases the risk for AD [4, 6-8].

**Figure 1.**
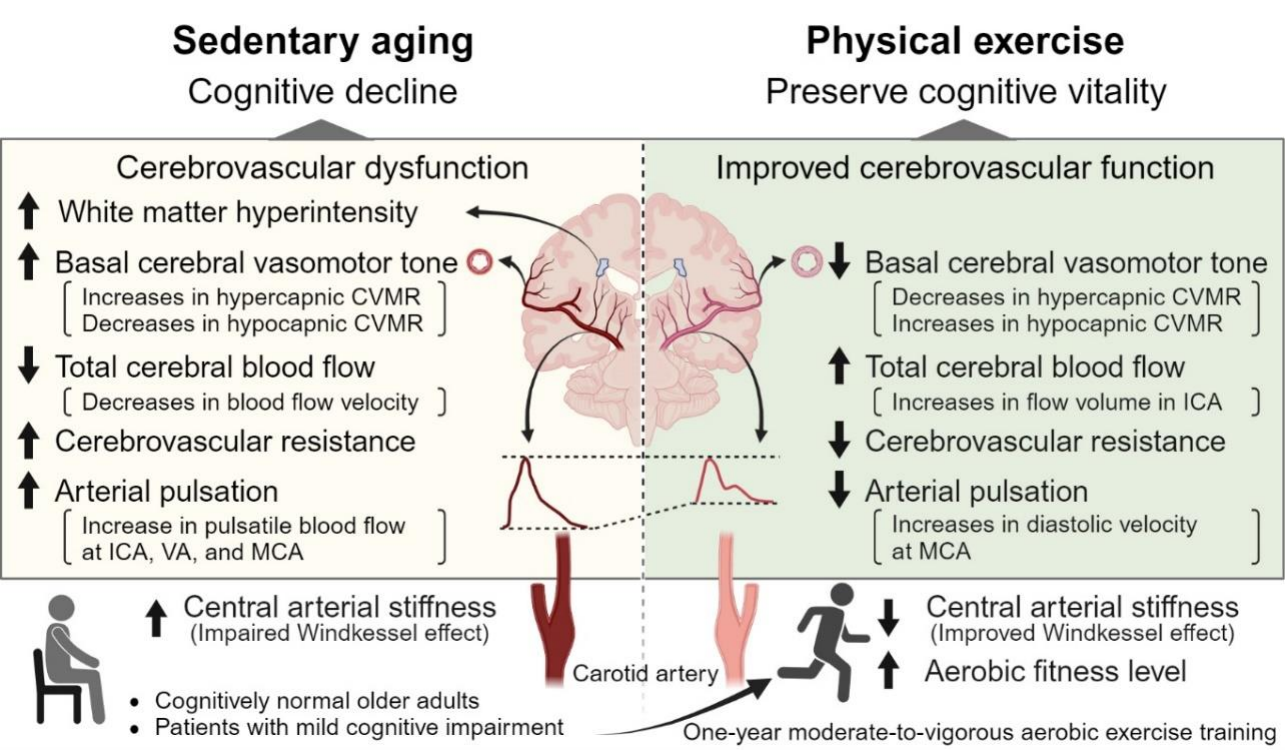
**A proposed hypothesis of arterial aging and cerebrovascular function in sedentary aging and physical exercise**. The Windkessel effect of central elastic arteries on cerebrovascular and cognitive function in aging and how aerobic exercise training may prevent or ameliorate the effects of arterial aging on cerebrovascular and cognitive function. CVMR, cerebral vasomotor reactivity; ICA, internal carotid artery; VA, vertebral artery; MCA, middle cerebral artery. Created with BioRender.com.

It has been hypothesized that age-related central arterial stiffening associated with a sedentary lifestyle increases systemic and cerebral arterial pulsation (i.e., pulsatile arterial pressure and/or blood flow) which may expose cerebral small blood vessels to augmented mechanical stress, thus leading to cerebral endothelial dysfunction, increase in cerebrovascular resistance (CVR), and cerebral hypoperfusion, and that these cerebrovascular dysfunctions may contribute to age-related cognitive decline or cognitive impairment related to AD (Fig. 1) [9-11]. Consistent with this hypothesis, increases in central arterial stiffness have been linked with the presence of cerebral small vessel disease (CSVD) manifested as magnetic resonance imaging (MRI) measurement of brain white matter intensities (WMH), cognitive impairment, and brain AD pathology (amyloid and tau depositions in older adults) [12-15]. In addition, further reduced cerebral blood flow (CBF), elevated central arterial stiffness, and cerebrovascular dysfunction have been observed in older adults with MCI compared with cognitively normal older adults [14-19].

Mounting evidence suggests that aerobic exercise training improves cerebrovascular function and thereby may prevent or slow age-related cognitive decline or the progression of AD [20-25]. Despite the recognized importance of exercise training/physical activity for preserving brain health, the underlying mechanisms are not well understood [20-26]. This lack of knowledge contributes to the uncertainty as to what type or dose of exercise (intensity, frequency, and duration) would influence exercise responses, who would get the most benefit from exercise and how exercise contributes to improvement in cerebrovascular function, thereby cognition function. In this regard, reductions of central arterial stiffness with aerobic exercise training have been observed in cognitively normal older population [27-29]. In this context, it has been proposed that a reduction in central arterial stiffness may decrease systemic and cerebral arterial pulsation and CVR and increase CBF, leading to preserved cognitive vitality in older adults (Fig. 1) [25].

The purpose of this brief review is to provide evidence that arterial aging, as manifested by increases in central arterial stiffness, and augmented arterial pulsation are associated with a reduction in CBF, cerebrovascular dysfunction, increases in brain WMH, and brain atrophy in older adults. Further, we provide evidence that one-year moderate-to-vigorous aerobic exercise training improves CBF which is associated with reductions in central arterial stiffness, arterial pulsation, and CVR and that improvement in cerebrovascular function is associated with improvement in cognitive function. Finally, we will discuss a potential dose-response relationship between changes in aerobic fitness level measured with peak oxygen uptake (VO_2peak_) with aerobic exercise training and reductions in central arterial stiffness and improvements in cerebrovascular and cognitive function.

There are several excellent systemic reviews and meta-analysis papers on exercise training, cerebrovascular function, and cognitive performance in older adults [20-25]. In this review, we will focus on the arterial aging hypothesis discussed above and provide supportive evidence based mainly on our previous studies of arterial aging across the adult lifespan [30-32] and aerobic exercise training in cognitively normal older adults and patients with MCI [33-35]. Conducting aerobic exercise training in patients with MCI is important because MCI may represent a critical time window for implementing lifestyle modifications to prevent further cognitive impairment [1, 5].

## Arterial Aging, Brain Structure, and Cerebrovascular Function

Advanced age is associated with the stiffening of central large elastic arteries which is a key determinant of augmented arterial pulsation and appears to lead to brain structural changes and cerebrovascular dysfunction [9-12, 15, 36]. Below, we will discuss the physiological role, assessment methods, and effects of central arterial stiffness on brain structural changes and cerebrovascular function as well as its association with cognitive decline.

### Central elastic artery stiffness and brain structure

The central elastic arteries (e.g., the aorta and carotid arteries) fulfill a physiological role in buffering arterial pulsations originated from the heart and provide continuous blood flow to the peripheral vascular beds, which is referred to as the Windkessel effect [37]. The main components responsible for buffering the mechanical stresses exerted on the arterial wall are elastin, collagen, and smooth muscle [36]. The central arterial wall elastin bears the vast majority of pulsatile mechanical stress generated from intermittent left ventricular ejection [36]. The central arterial wall expands to accommodate stroke volume during systole, which attenuates the transmission of excessive systolic pressure energy into the downstream microcirculation [10, 37]. During diastole, the arterial wall recoils due to stored energy to maintain diastolic blood pressure (BP) and blood flow to the peripheral vascular beds [37]. The Windkessel effect of the central elastic artery protects the key end-organs (e.g., the brain and kidney) from being subjected to potentially damaging excessive arterial pulsation while preserving the efficiency of tissue perfusion [10, 36].

A number of methodologies have been used to assess the elastic properties of central arteries in humans [36]. The carotid-femoral pulse wave velocity (cfPWV), which has been considered as the gold standard for the measurement of central arterial stiffness, is determined by the distance from the carotid to the femoral arteries and the time taken for the arterial pulse wave to propagate between the two sites [38]. Consequently, cfPWV assesses an integrated stiffness of different segments of the aorta [36]. Alternatively, carotid arterial stiffness (e.g., the carotid β-stiffness index) is determined by the measurements of lumen diameter changes using ultrasound imaging and the arterial pulse pressure via applanation tonometry recorded at the common carotid artery, which is a regional arterial stiffness measure close to the brain [36]. In addition, compared to cfPWV, measurement of carotid arterial stiffness is less influenced by changes in arterial pressure [39]. Thus, carotid arterial stiffness is likely to be more relevant and reliable to assess the impacts of central arterial stiffening on the brain [32, 40].

Age-related central arterial stiffening can be attributed to elastin fragmentation, collagen deposition, and altered vascular smooth muscle tone [36]. The central arterial stiffening impairs the Windkessel effect, which may lead to increases in pulsatile arterial pressure and blood flow, thereby damaging the small blood vessels in the brain [9, 11]. The brain is vulnerable to arterial pulsation because it has low vascular resistance and high perfusion, thus elevated arterial pulsation may penetrate downstream into the microcirculation causing CSVD [9, 11]. CSVD manifested as WMH is closely associated with age-related brain atrophy and cognitive decline [41, 42]. Indeed, higher central arterial stiffness assessed by cfPWV has been associated with the elevated pulsatile arterial pressure, greater WMH volume, brain atrophy, and cognitive decline in the elderly with or without cognitive impairment [12, 14, 15].

To gain the insights into the association of age-related central arterial stiffening, in particular carotid arterial stiffness, with brain structural changes across the adult lifespan, we recently studied the associations of central arterial stiffness measured by cfPWV and carotid arterial stiffness with brain volume and WMH in 187 healthy adults aged between 21 and 80 years [32]. The participants in this study were vigorously screened for the presence of clinical cardiovascular disease and/or cardiovascular risk factors related to central arterial stiffness. In particular, those with BP ≥ 140/90 mmHg, consolidated with 24-hour ambulatory BP monitoring, were excluded because hypertension has a significant impact on central arterial stiffness [32]. We found that cfPWV increased linearly while carotid arterial stiffness increased nonlinearly with advanced age although both measures of central arterial stiffness were highly correlated (*R^2^* = 0.40). However, this correlation was weakened among people aged more than 46 years (*R^2^* = 0.15) suggesting a divergence of these two measures in advanced age [32]. In addition, CBF pulsatility at the middle cerebral artery (MCA) measured by transcranial Doppler (TCD) and WMH volume increased, whereas total brain and gray matter volumes decreased with age, consistent with previous meta-analysis and systematic reviews [12, 14, 15].

**Figure 2.**
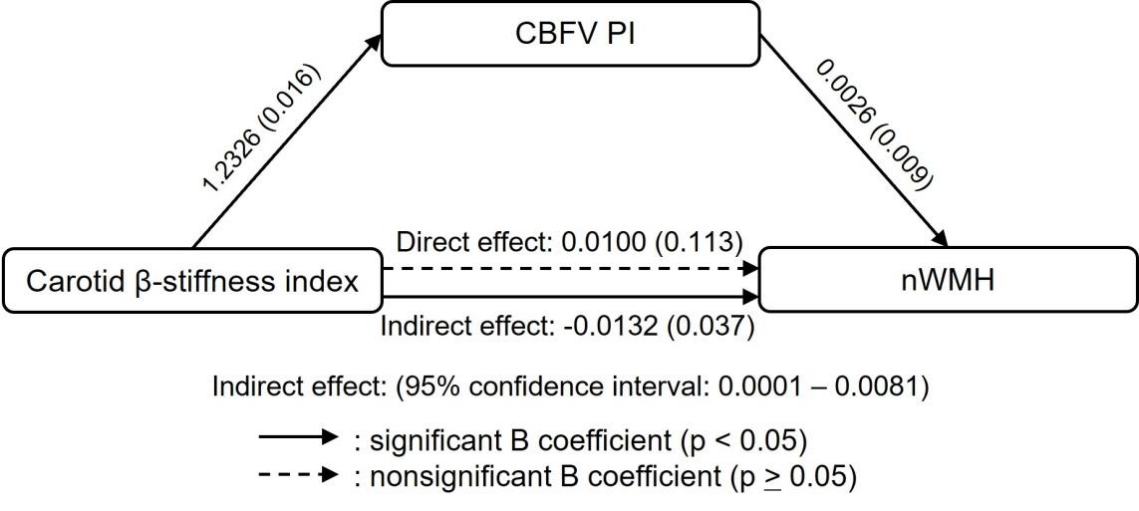
**A mediation analysis of the relationship between carotid β-stiffness index, normalized white matter hyperintensity (nWMH) by individual brain volume, and cerebral blood flow velocity pulsatility index (CBFV PI)**. B, unstandardized regression coefficients. One-hundred fifty-nine subjects aged between 21 and 80 years were used for modeling. Covariates included in the mediator model: age, age^2^, sex, and mean arterial pressure. [Adapted from [32] Copyright © (2023) with permission from Wolters Kluwer Health Inc.

This study extends previous investigations by revealing the associations of both age-related carotid arterial stiffness and cfPWV with brain structural alterations across the adult lifespan [32]. Higher carotid arterial stiffness and cfPWV were associated with larger WMH volume, and higher cfPWV was associated with smaller total brain volume and gray matter volume after adjustment of age, sex, and mean arterial pressure. Notably, we observed that CBF pulsatility mediated the associations between the increase in carotid arterial stiffness and WMH volume after adjustment for age, sex, and mean arterial pressure (Fig. 2) [32]. Collectively, these findings suggest that the Windkessel effect of the central artery on buffering arterial pulsation is related to brain structural changes in normal aging[19].

### Cerebral blood flow and cerebrovascular resistance

A sufficient and continuous blood supply of oxygen, nutrients, and energy substrates (i.e., glucose) to the brain is necessary to maintain normal neuronal function [4, 6, 43]. In human, the brain represents only 2-3% of total body mass while requiring ~15% of cardiac output and consuming about ~ 20% of the available O_2_ under normal conditions [4, 6, 43]. The high metabolic rate of the brain, combined with limited energy stores, highlights the importance of CBF for nutrients and O_2_ delivery [4, 6, 43]. To sustain the high-volume blood supply, CVR is low relative to the other organs [43]. Importantly, a large part of CVR is controlled outside of the parenchyma by the cerebral arteries and pial arterioles [6, 43]. Thus, a normal function of CVR adjustment in response to the blood flow demand is crucial in maintaining normal brain function.

A number of non-invasive imaging modalities have been used to measure volumetric CBF, CBF velocity, and brain perfusion [44]. For example, phase-contrast magnetic resonance imaging (PC-MRI) and color-coded duplex ultrasonography (CDUS) have been used to measure both volumetric CBF and CBF velocity at the brain-feeding extracranial arteries [i.e., the internal carotid (ICA) and vertebral (VA) arteries]. Furthermore, TCD has been used widely to measure CBF velocity at the intracranial arteries [e.g., the MCA] to reflect changes in CBF. Finally, an MRI arterial spin labeling (ASL) approach has been used to measure both global and regional brain perfusion.

In most of the previous studies, age-related changes or differences in CBF were often measured by using only one of the aforementioned modalities [45, 46]. It is therefore difficult to compare these studies directly because of the different methods used for CBF measurement. We recently reported the measurement of CBF using multimodality approaches in healthy adult population [30]. We observed that measurements of total CBF and normalized CBF by individual brain volume were correlated among CDUS, PC-MRI, and ASL. The measurements of blood flow velocity at the ICA, VA, and MCA were also correlated among CDUS, PC-MRI, and TCD despite the presence of large individual differences which may reflect either the individual physiological variabilities or the methodological differences or both [30].

Age-related reduction in CBF has been reported in previous studies, which may reflect either a reduction of cerebral metabolism, cerebrovascular dysfunction, or both [43, 45, 46]. One hypothesis in support of cerebrovascular dysfunction is that age-related central arterial stiffening associated with increases in arterial pulsation may expose cerebral arterioles and capillaries to augmented mechanical stress, thus leading to cerebral endothelial dysfunction, vasoconstriction, increases in CVR, and decreases in CBF in older adults (Fig. 1) [9, 11]. Of note, a longitudinal study in older adults with and without clinical diagnosis of AD showed that increases in CVR preceded reductions in CBF and that increases in CVR were able to predict the onset of clinical AD independent of alterations of cerebral metabolism [47]. These observations are consistent with a recent report that cerebrovascular dysfunction manifested as brain hypoperfusion precedes the development of AD pathology in older adults [18].

To investigate the CBF and CVR across the healthy adult lifespan, we studied the age-related differences in CBF and CVR using MRI, ultrasonography, and TCD in 185 healthy adults aged between 21 and 80 years [30]. In this study, CBF velocity and the vessel diameters of the ICA and VA were simultaneously measured to determine whether age-related differences in CBF are determined mainly by the alternations in the blood flow velocity or the vessel diameters, or both. Since large cerebral arteries such as the ICA and VA contribute importantly to the overall CVR, these measurements also provide insight into whether age-related increases in CVR can be attributed mainly to the vasoconstrictions of the downstream small cerebral arterioles and/or capillaries [4, 43].

**Figure 3.**
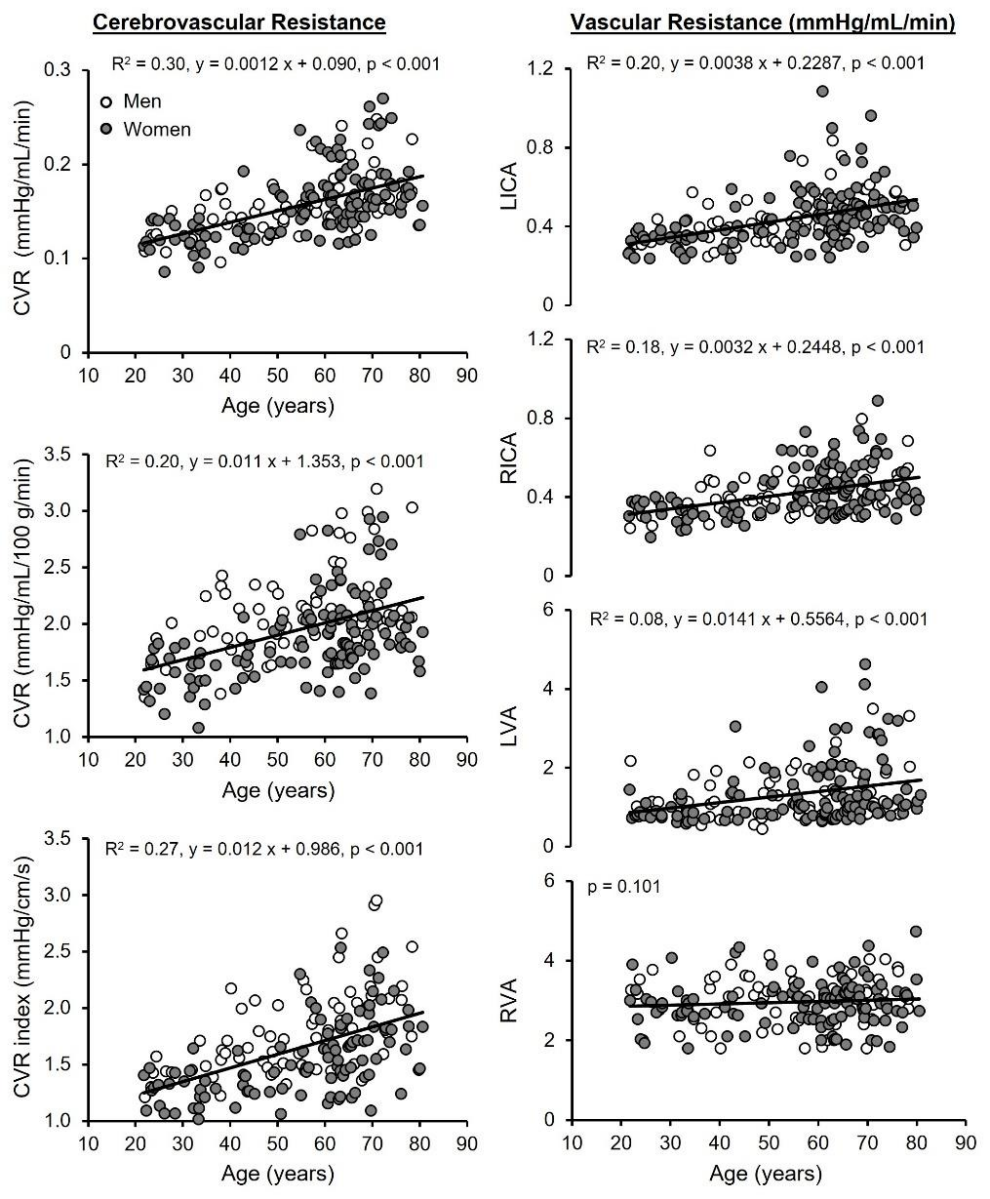
**Association of age with cerebrovascular resistance (CVR)**. CVR measured by color-coded duplex ultrasonography (left upper and middle panels), CVR index measured by transcranial Doppler at the middle cerebral artery (left lower panel), and vascular resistance in the left (L) and right (R) internal carotid (ICA) and vertebral arteries (VA) (right panels) are shown. CVR was calculated as mean arterial pressure (MAP) divided by total cerebral blood flow (CBF) (left upper panel) and normalized CBF (left middle panel). Vascular resistance was calculated as MAP divided by blood flow volume in each artery. Solid lines represent the regression equations obtained for 185 subjects in CVR and vascular resistance and 169 subjects in CVR index. [Adapted from [30] Copyright © (2023) with permission from SAGE Publications.

We found that age was associated with decreased CBF by ~3.5 mL/min per year and CBF normalized by individual brain volume by ~0.19 mL/100 g/min per year across the measurement methods used, and that these magnitudes of reductions in CBF with age are consistent with previous studies [45, 46]. Of note, similar to other studies of age-related differences in CBF, we cannot dissect other confounding factors that may influence CBF such as individual differences in brain metabolic rate or medication use in older adults (e.g., antihypertensives or cholesterol medication). In this regard, recent studies suggest that the effects of antihypertensives or statins on CBF in otherwise healthy older adults are likely to be minimal [48-50].

We also observed that CVR increased by ~0.011 mmHg/mL/100 g/min per year and vascular resistance measured at the ICAs and VAs also increased with age (Fig. 3) [30]. Blood flow velocities measured at the ICAs, VAs, and MCA decreased linearly with age ranging from 0.07 - 0.15 cm/s per year, while the vessel diameters of the ICAs and VAs remained similar among the age groups. Furthermore, increases in CBF pulsatility at the ICAs, VAs, and MCA with age were also observed [30]. Collectively, these results suggest the presence of cerebral vasoconstriction which likely occurs in the small cerebral arterioles and capillaries but not in the large cerebral arteries [30]. These observations are also consistent with the hypothesis that reduction of downstream blood flow velocity may reduce the shear stress on the blood vessel endothelial cells and flow-mediated vasodilation leading to increases in CVR and reductions in CBF, which in turn may formulate a vicious circle affecting neuronal function [11, 43].

There is an increasing recognition that cerebral hypoperfusion, increased CVR, and central arterial stiffening are emerging risk factors for clinical AD [36]. We recently tested this hypothesis by showing that carotid arterial stiffness is associated with reduced CBF, and increased CVR in patients with MCI [40]. Patients with MCI had lower CBF and higher CVR when compared with age-matched cognitively normal older adults [40, 51]. Importantly, CBF was negatively associated with carotid arterial stiffness, and CVR was positively associated with carotid systolic pressure after adjustment for age, sex, body mass index, and MCI status [40]. Furthermore, CBF pulsatility measured at the MCA was positively associated with carotid pulse pressure and negatively with diastolic BP. Of note, lower diastolic CBF velocity at the MCA was also associated with higher carotid arterial stiffness and lower CBF, suggesting that impaired Windkessel effect during diastole may contribute to the overall reduction in CBF in patients with MCI [40]. Alternatively, the presence of AD pathology such as brain β-amyloid and tau in patients with MCI may cause cerebral vasoconstriction leading to reductions in CBF and increases in CVR [52, 53].

### Cerebral vasomotor reactivity to CO_2_ during hypo- and hypercapnia

CBF is highly sensitive to changes in the partial pressure of carbon dioxide in the arterial blood (PaCO_2_). Elevated PaCO_2_ (hypercapnia) increases CBF via cerebral vasodilation, whereas reduced PaCO_2_ (hypocapnia) decreases CBF due to vasoconstriction [54, 55]. These CBF responses to changes in PaCO_2_ are referred to as cerebral vasomotor reactivity (CVMR), which can be assessed during either hypercapnia or hypocapnia, or both [54, 55]. The changes in cerebral vasomotor tone to PaCO_2_ may occur throughout the cerebrovascular tree but likely occur mainly in the small cerebral arterioles and the capillary vascular beds [4, 43]. Although the underlying molecular and cellular mechanisms of CVMR to changes in arterial CO_2_ are not well understood, it may reflect cerebral blood vessels’ responses to neuronal metabolic stimuli, thus neurovascular coupling (NVC) [6, 56]. Accordingly, the measurement of CVMR has been used widely in clinical and research settings to assess cerebrovascular function [57]. However, it should be mentioned that NVC can be assessed directly by measuring CBF responses to cognitive stimuli (e.g., measurement of changes in CBF velocity using TCD during memory/executive testing) [58-60]. Whether the measurement of CVMR is correlated with direct measurement of NVC and whether the underlying molecular and cellular mechanisms leading to cerebral vasodilation and increase in CBF are different or similar between these assessments need to be determined in future studies [56, 60, 61].

Several methods are available to assess CVMR during either hyper- or hypocapnia [57]. CVMR during hypercapnia can be assessed either by using stepwise increases in inspiratory air concentration of CO_2_ [54, 55] or a rebreathing method in which a progressive increase in PaCO_2_ was induced by having the subject rebreathe his/her own expired air [62]. Similar results of CVMR measurements between the two methods using TCD have been reported previously [63]. On the other hand, CVMR during hypocapnia is commonly assessed by asking the study participants to perform a short period of hyperventilation of room air to induce progressive decreases in PaCO_2_ [63-65]. It has been reported that measurement of CVMR was influenced by the marked changes in systemic arterial BP during hypo- and hypercapnia which are likely mediated by the central and peripheral chemoreceptor responses to change in PaCO_2_ [66]. Thus, it is essential that changes in systemic arterial BP need to be accounted for the changes in CBF during CVMR assessment [43].

The reduction of hypocapnia CVMR during hyperventilation (cerebral vasoconstriction) has been observed in older adults either with or without cognitive impairment [64, 65, 67]. These studies suggested that cerebral vasoconstrictor capacity is reduced in older adults compared with young individuals. Furthermore, previous studies using TCD during voluntary hyperventilation reported lower hypocapnic CVMR in patients with AD and vascular dementia [67], but not in MCI [68].

In contrast, the effects of advanced age on hypercapnic CVMR (cerebral vasodilation) are inconsistent [69-71]. Hypercapnic CVMR using the steady-state (i.e., stepwise increases in inspiratory air concentration of CO_2_) and breath-holding techniques have reported a reduction or no change with age [69, 70]. Conversely, we observed that hypercapnic CVMR was enhanced in cognitively normal older adults compared with young individuals in a small sample size [71]. The findings of hypercapnic CVMR in patients with AD and MCI when compared with cognitively normal older adults are also inconsistent [16]. These discrepancies may reflect the limitations of the relatively small sample size employed in these studies, the differences in the methodologies used to measure CBF, the statistical modeling and data analysis of the vascular responses, and the magnitude of manipulated changes in PaCO_2_. Especially, most of these studies did not account for the changes in systemic arterial BP during changes in PaCO_2_ which may have contributed to the observed inconsistent results [43].

Given the limitations in CVMR assessments mentioned above, we recently studied CVMR to CO_2_ during both hypo- and hypercapnia across the adult lifespan in 153 healthy adults aged between 21 and 80 years [31], and in 70 patients with MCI [72] using the same hyperventilation and rebreathing methods [62, 63]. During both the hypo- and hypercapnic protocol, we measured breath-by-breath changes in end-tidal CO_2_ and beat-by-beta changes in CBF velocity at the MCA via TCD and finger arterial BP with non-invasive approaches to account for changes in BP on the assessment of CVMR [31, 72].

**Figure 4.**
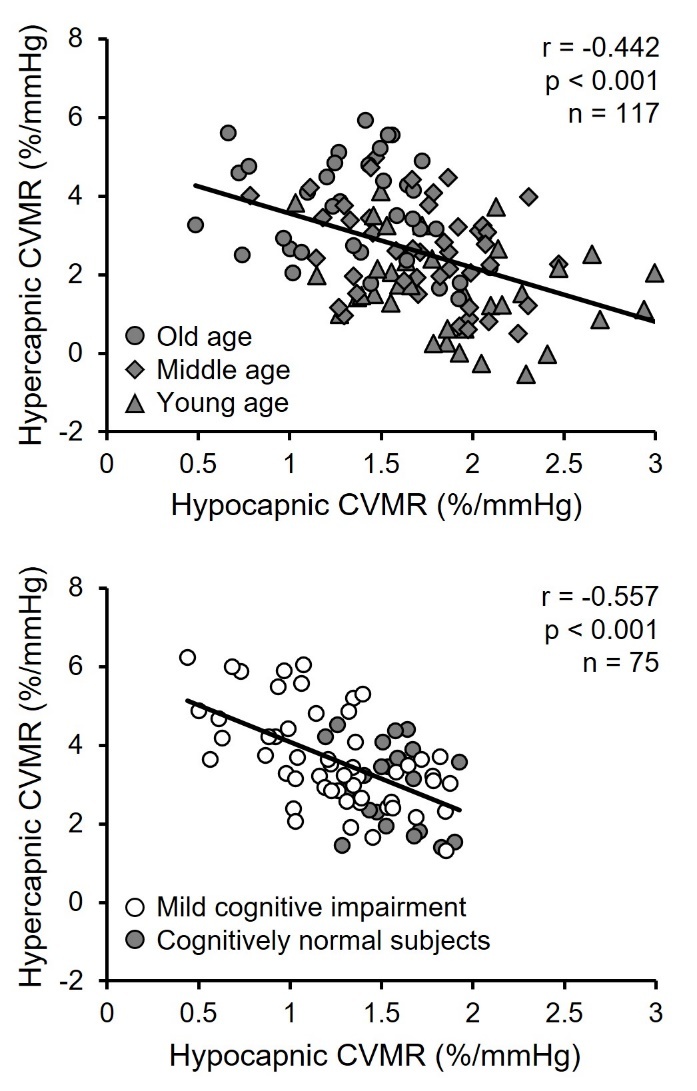
**Simple correlations between hypo- and hypercapnic cerebral vasomotor reactivity (CVMR)**. The correlations across the adult lifespan aged between 21 and 80 years are shown in upper panel and among cognitively normal older adults and patients with amnestic mild cognitive impairment shown in lower panel. CVMRs were calculated from the slope of cerebrovascular conductance index (CVCi, %) vs. end-tidal CO_2_ (mmHg). CVCi was calculated as mean cerebral blood flow velocity (CBFV) at the middle cerebral artery divided by mean arterial pressure (MAP). CVCi was used to account for the effects of changes in MAP on CBFV during hypo- and hypercapnia. [Upper panel adapted from [31] Copyright © (2020) with permission from SAGE Publications and lower panel adapted from [72] Copyright © (2020), with permission from IOS Press.

Several important results were observed in these studies [31, 72]. First, we observed that hypocapnic CVMR was reduced while hypercapnic CVMR was increased with age [31]. Second, patients with MCI had lower hypocapnic CVMR, but higher hypercapnic CVMR compared with cognitively normal older adults [72]. Third, hypo-and hypercapnic CVMR were inversely correlated to each other across all subjects (Fig. 4) [31, 72]. Fourth, BP response to hypercapnia was augmented with advanced age and in patients with MCI. We also observed that lower hypocapnic CVMR and higher hypercapnic CVMR were associated with lower performance scores of episodic memory and executive function in cognitively normal older adults and patients with MCI [72].

Our observation of an inverse relationship between hypo- and hypercapnic CVMR in Figure 4 provides further evidence in support of the presence of cerebral vasoconstriction and increases in CVR with advanced age and in patients with MCI. We hypothesized that increases in the basal cerebral vasomotor tone (vasoconstriction) with age and in patients with MCI may shift the baseline operating point of the PaCO_2_-CBF relationship downward closer to the cerebral ischemic threshold which would decrease the hypocapnic cerebral vasoconstriction reserve [71]. On the other hand, a downward shift of the operating point may result in a greater reserve for cerebral vasodilation, consistent with the observed inverse relationship between hypo- and hypercapnic CVMR presented in Figure 4. Collectively, these observations support our central hypothesis that cerebrovascular dysfunction may occur with advanced age and accelerate in the early phase of clinical AD contributing to cognitive decline (Fig. 1).

## Effects of Aerobic Exercise Training on Vascular and Brain Health

Interventions targeting to reduce central arterial stiffness have a potential to improve cerebrovascular function and reduce the risk of AD [7, 36]. Aerobic exercise training reduced central arterial stiffness has been reported in previous studies [20, 25, 27, 29]. However, at present, the effects of aerobic exercise training on improving cerebrovascular function and cognitive performance in older adults are inconclusive [73, 74]. Below, we will discuss the relationship between exercise-induced reduction in central arterial stiffness and improvement in cerebrovascular and cognitive function in older adults based mainly on our previous studies [33-35].

### Central elastic artery stiffness, cerebral blood flow, and cognitive function

It has been hypothesized that regular aerobic exercise decreases age-related stiffening of the central elastic artery in older adults [27-29], which may lead to increases in CBF [20, 25]. In this regard, previous studies of 3 - 4 months of aerobic exercise training reduced central arterial stiffness in older adults and these observations have been interpreted to suggest that the reduced central arterial stiffness with exercise training reflects mainly a reduction in the vascular smooth muscle tone because the elastin-collagen compositions of the central arterial wall, which represent a major component of arterial stiffness, are unlikely to be modified with short-term aerobic exercise training [29]. It has been well established that arterial smooth muscle tone is modulated mainly by the vessel wall endothelial function related to nitric oxide bioavailability [27, 29]. Thus, it is possible that exercise-induced reduction in central arterial stiffness, arterial pulsation, and improvement in cerebral endothelial function may lead to decreases in CVR and increases in CBF [25, 43]. In addition, exercise-induced increases in capillary density [75] and/or increases in cerebral metabolic rate of oxygen may also lead to increases in CBF through NVC [21].

The effects of aerobic exercise training on CBF are inconclusive [21, 23, 24]. A recent systematic review and meta-analysis of the effects of cardiorespiratory fitness and aerobic exercise training on CBF reported that higher cardiorespiratory fitness was associated with higher CBF velocity measured at the MCA using TCD among older adults in cross-sectional studies [24]. However, moderate intensity of aerobic exercise training for a duration of 2-12 months had little influence on the MCA CBF velocity and global cerebral perfusion measured using MRI ASL [23, 24]. One of major limitations in measuring changes in CBF using TCD is that changes in CBF velocity do not necessarily equal changes in volumetric CBF [57]. In addition, measurements of global cerebral perfusion using ASL are limited by low signal/noise ratio, particularly in the white matter, and some of arbitrary model parameter assumptions used to calculate CBF (e.g., post labeling delay) which may be altered by engaging in exercise training [76]. In this regard, global CBF measured as the sum of volumetric blood flow from both the ICA and VA using 2D CDUS may overcome the limitations of global CBF measurements using TCD and MRI ASL [30].

To gain insights into the effects of aerobic exercise training on CBF, CVR, and cognitive performance, we conducted one-year, open-label, paralleled randomized control trials in both cognitively normal older adults [35] and patients with MCI [33]. The effects of moderate-to-vigorous aerobic exercise training on CBF, CVR, and cognitive performance were compared with an active control group of stretching-and-toning interventions.

**Figure 5.**
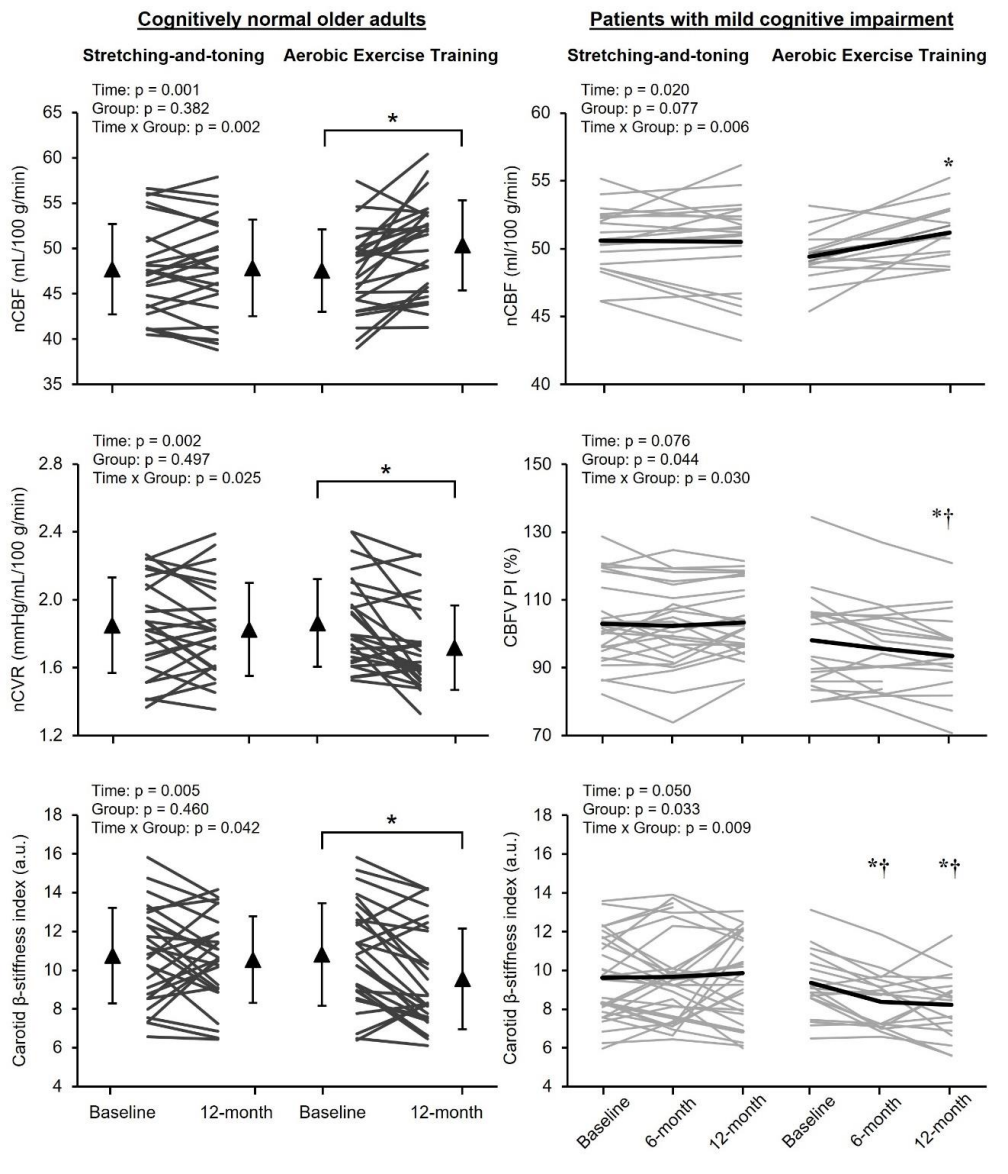
**Effects of aerobic exercise training on central arterial stiffness and cerebrovascular function**. Each panel shows changes in normalized cerebral blood flow (nCBF) and normalized cerebrovascular resistance (nCVR) by individual brain volume, CBF velocity pulsatility index (CBFV PI) at the middle cerebral artery, and carotid β-stiffness index after one-year stretching-and-toning or aerobic exercise training. Thin lines represent individual changes in all panels. Triangles show mean values, and the error bars represent standard deviations (left panels). The thick line represents the estimated marginal means with linear mixed model analysis. * p < 0.05 compared with baseline after Bonferroni correction. † p < 0.05 compared with the SAT group. [Left panels adapted from [35] Copyright © (2022) with permission from SAGE Publications, and right panels adapted from [33] Copyright © (2021), with permission from IOS Press.

**Figure 6.**
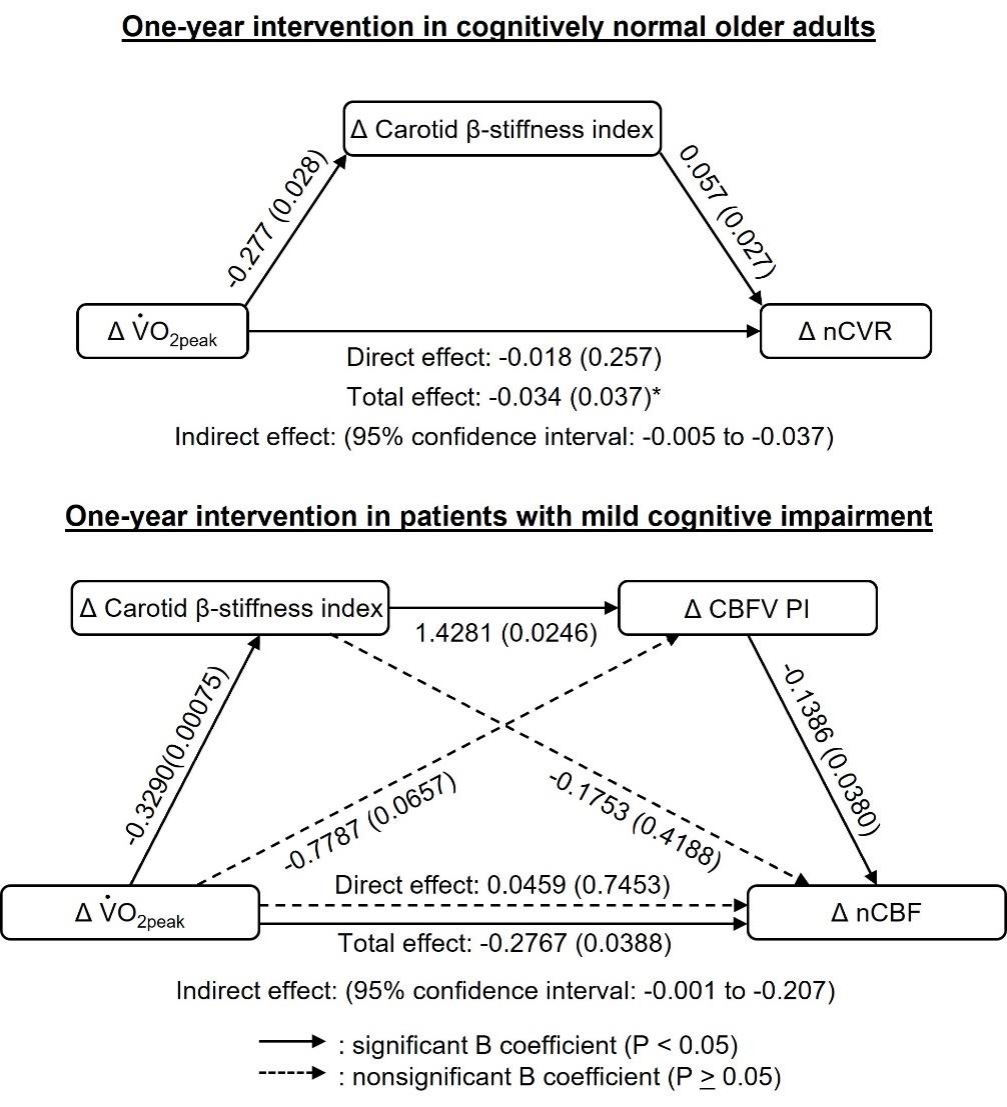
**Mediation analyses of the relationship between changes in aerobic fitness level and cerebrovascular function with central arterial stiffness as a mediator**. Mediation analyses show the relationship between changes in peak oxygen uptake (VO_2peak_) and normalized cerebrovascular resistance with carotid β-stiffness index as a mediator in the aerobic exercise training group in one-year intervention in cognitively normal older adults (upper panel n = 28), and the relationship between changes in VO_2peak_ and normalized cerebral blood flow (nCBF) with carotid β-stiffness index and CBF velocity pulsatility index (CBFV PI) as mediators across the groups in the intervention in patients with amnestic mild cognitive impairment (lower panel n = 30). [Upper panel adapted from [35] Copyright © (2022) with permission from SAGE Publications and lower panel right panels adapted from [33] Copyright © (2021), with permission from IOS Press.

We found that the one-year aerobic exercise training increased global CBF and decreased CVR and carotid arterial stiffness in both cognitively normal older adults and in patients with MCI (Fig. 5) [33, 35]. Of note, aerobic exercise-induced increases in CBF were due mainly to the increased ICA blood flow in both cognitively normal older adults and in patients with MCI [33, 35]. Furthermore, we found that reduced carotid arterial stiffness was associated with increased CBF in both cognitively normal older adults and patients with MCI and decreased CVR in cognitively normal older adults [33, 35]. The mediation analysis showed that the negative associations between changes in VO_2peak_ and CVR were mediated by the reduction of carotid arterial stiffness in cognitively normal older adults (Fig. 6) [35]. In the patients with MCI, CBF pulsatility was reduced in the aerobic exercise group (Fig. 5) and a mediation analysis showed that the positive associations between change in VO_2peak_ and CBF were mediated by reductions in carotid arterial stiffness and CBF pulsatility (Fig. 6) [33].

We found that cognitive performance, mainly memory function, was improved slightly but significantly after one year of exercise intervention in cognitively normal older adults [33, 35]. Specifically, aerobic exercise training-induced reduction in carotid stiffness and CVR were associated with improved Woodcock-Johnson immediate recall scores [35]. However, aerobic exercise training did not prevent reduction in brain volume in cognitively normal older adults and patients with MCI [33, 35]. These observations suggest that aerobic exercise training-induced improvements in cerebrovascular function may precede changes in brain structure in older adults.

### Cerebral vasomotor reactivity to CO_2_ and cognitive function

Regular aerobic exercise training may improve CVMR in older adults [21, 24]. Besides, altered CVMR has been observed in patients with MCI and was associated with cognitive performance [72]. In this regard, moderate-to-vigorous intensity aerobic exercise training in a time frame of 3-6 months also improved peripheral endothelial function in older adults [73, 77, 78]. In a study of stroke survivors, 6-month of moderate-intensity of aerobic exercise training increased hypercapnic CVMR [77], and in healthy older adults, 3-month of moderate-intensity aerobic exercise also increased CVMR during 5% CO_2_ inhalation [78]. However, 6-month moderate-intensity aerobic exercise in cognitively normal middle-aged and older adults did not alter CVMR during hypercapnia [73]. These inconsistent findings likely reflect the differences in the study population, the exercise duration, as well as the methods used to measure CVMR.

**Figure 7.**
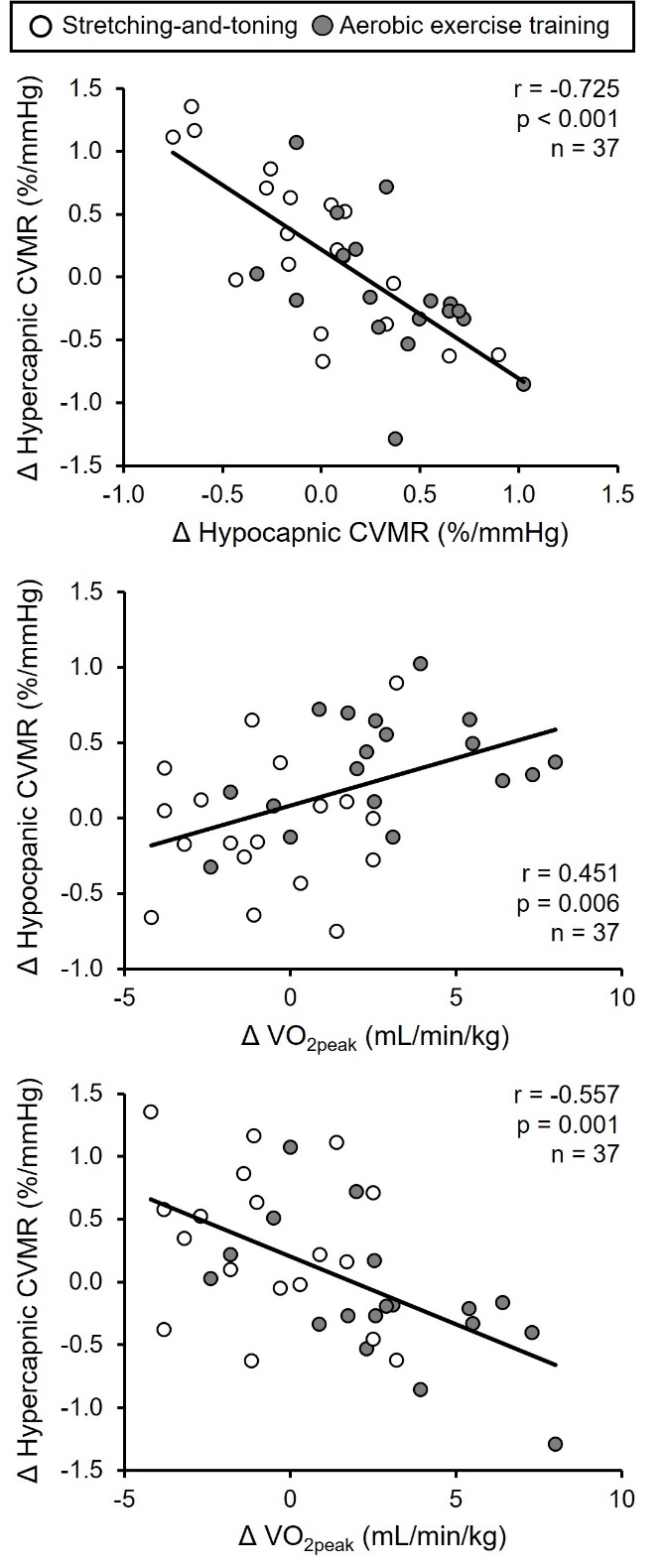
**Linear correlation between changes in hypocapnic and hypercapnic cerebral vasomotor reactivity (CVMR) and changes in peak oxygen uptake (VO_2peak_)**. CVMRs were calculated from the slope of cerebrovascular conductance index (CVCi, %) vs. end-tidal CO_2_ (mmHg). CVCi was calculated as mean cerebral blood flow velocity (CBFV) at the middle cerebral artery divided by mean arterial pressure (MAP). CVCi was used to account for the effects of changes in MAP on CBFV during hypo- and hypercapnia. Δ represents changes in pre- and post-interventions. [Adapted from [34] Copyright © 2021 with permission from the American Physiology Society.

We studied the effect of one-year aerobic exercise training on CVMR in patients with MCI during hypo- and hypercapnia [34]. We found that hypocapnic CVMR increased whereas hypercapnic CVMR decreased with aerobic exercise training when compared to stretching-and-toning [34]. Of note, changes in hypo- and hypercapnic CVMR were negatively correlated with each other, consistent with those presented above in Figure 4 of the cross-sectional studies of CVMR with aging and MCI (Fig. 7). We also observed that decreases in hypercapnic CVMR with aerobic exercise were correlated with improved cognitive performance in memory and executive function [34]. Collectively, these results suggest that one-year aerobic exercise improved CVMR which is associated with improvement in cognitive function in patients with MCI. The observed increases in hypocapnic CVMR and decreases in hypercapnic CVMR with aerobic exercise training may be related to the exercise-induced reduction of the basal cerebral vasomotor tone and CVR (i.e., baseline vasodilation before CO_2_ stimuli) in that cerebral vasoconstriction reserve during hypocapnia increases whereas cerebral vasodilation reserve during hypercapnia decreases, consistent with the observation that aerobic exercise improved endothelial function and flow-mediated vasodilation at rest [27].

It must be acknowledged that the salutary effects of aerobic exercise training on brain health are likely to be multifactorial and go beyond those of aforementioned changes in cerebral hemodynamics [21, 43, 79]. For example, previous studies showed that one-year aerobic exercise training increased hippocampal volume which was associated with greater serum level of brain derived neurotrophic factor (BDNF) and improvement in memory performance [79]. Further, potential anti-inflammatory effects of aerobic exercise training on improvement of cognitive function and cerebrovascular health have been proposed [21]. Further research is warranted to better understand the role of different biological/physiological mechanisms for the benefits of aerobic exercise training on brain health in older adults.

### The dose-response relationship between aerobic exercise training and improvement in cerebrovascular and cognitive function

Epidemiological studies indicated the presence of a dose-response relationship between physical activity/exercise training and the overall health benefits [80], and that this dose-response relationship may also apply to cognitive outcomes in that individuals who have greater levels of physical activity also have higher levels of cognitive performance and lower dementia risk [81, 82]. Of note, a community-based exercise training study by Vironi et al. showed that it was the magnitude of changes in cardiorespiratory fitness rather than the dose of exercise administrated during the intervention that was a better predictor of cognitive benefit in older adults [83]. Our previous studies in Masters athletes, those who have engaged in life-long high-intensity aerobic exercise training, also showed that life-long exercise may attenuate age-related brain tissue loss [84] and that VO_2peak_, which was significantly higher in Masters athletes than their age-matched sedentary controls, was correlated negatively to brain WMH volume [85].

**Figure 8.**
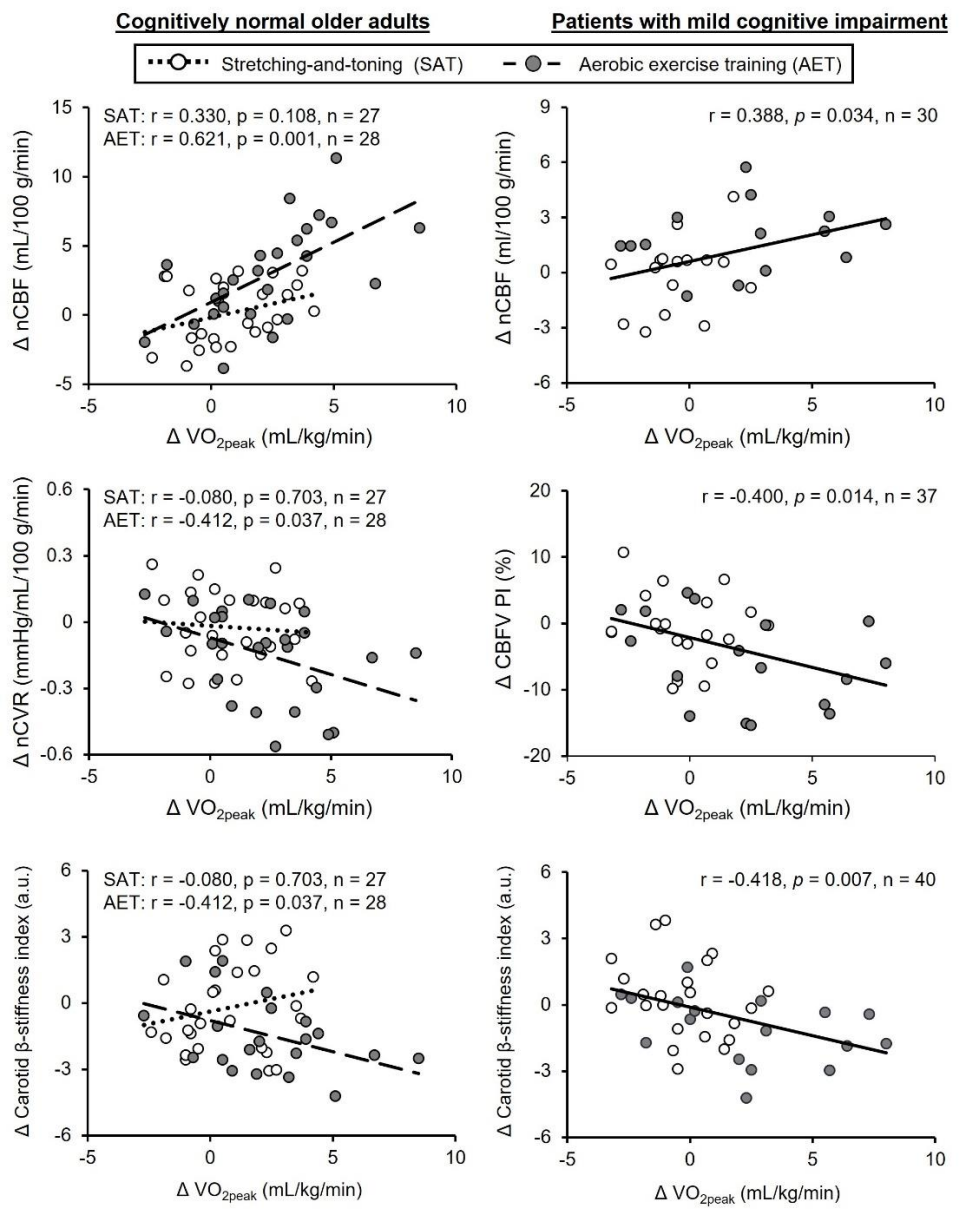
**Associations of changes in aerobic fitness level with central arterial stiffness and cerebrovascular function**. Correlations of one-year changes in peak oxygen uptake (VO_2peak_) with normalized cerebral blood flow (nCBF) and cerebrovascular resistance (nCVR) by individual brain volume, carotid β-stiffness index, and cerebral blood flow pulsatility index (CBFV PI) at the middle cerebral artery were reported separately in cognitively normal older adults (left panels but across in the patients with mild cognitive impairment (right panels). Solid line, dotted line, and broken line represent those obtained from all, aerobic exercise training, and stretching-and-toning, respectively. [Left panels adapted from [35] Copyright © (2022) with permission from SAGE Publications, and right panels adapted from [33] Copyright © (2021), with permission from IOS Press.

In this regard, a dose-response relationship between aerobic exercise training and improvement in cerebrovascular function may also present [21, 80, 86]. It has been proposed that the benefits of aerobic exercise training on CBF may manifest only when VO_2peak_ is significantly improved [21, 86]. For example, a single-arm, 6-month moderate-to-vigorous aerobic exercise training increased cardiorespiratory fitness by 8% and slightly but significantly increased CBF velocity measured at the MCA in cognitively normal older adults [73]. Further, a 3-month moderate-to-vigorous aerobic exercise training increased VO_2peak_ by 6% and increased regional cerebral perfusion measured with ASL in the anterior cingulate cortex compared with the control group in healthy older adults [87]. Furthermore, 4-month moderate-to-vigorous aerobic exercise training increased cerebrovascular responses to cognitive stimuli in older adults, and the magnitude of improvements in cerebrovascular function was associated positively with the amount of exercise sessions performed [59].

Consistent with previous studies, we observed one-year progressive moderate-to-vigorous aerobic exercise training increased VO_2peak_ by ~9% and increased global CBF by ~4%, and decreased CVR by ~5% measured with ultrasonography in patients with MCI relative to baseline [33]. In cognitively normal older adults, one-year progressive moderate-to-vigorous aerobic exercise training increased VO_2peak_ by ~10%, global CBF by ~5%, and decreased CVR by 7% [35]. Of note, we also observed that increases in VO_2peak_ were associated with increased CBF and reduction in CVR and carotid arterial stiffness in cognitively normal older adults and patients with MCI (Fig. 8) [33, 35]. Furthermore, mediation analyses showed that the effects of changes in VO_2peak_ on CBF and CVR were mediated by changes in exercise-induced improvement of carotid arterial stiffness (Fig. 6) [33, 35].

## Clinical Implications

Dementia is one of the biggest social and scientific challenges in the 21^st^ century [1, 22]. Besides its significant impact on the health-related quality of life of the affected individuals and their family members, the financial burden to the patients, their families, and the society at large is huge if not unbearable [1]. The clinical effects of recently developed anti-amyloid therapy for AD, the most common type of dementia, were modest and the long-term outcome is unknown [3]. Therefore, development of effective interventions for dementia prevention is essential for maintaining cognitive vitality in late life.

In this review, we showed that arterial aging manifested by increases in central arterial stiffness is associated with reduction in CBF, altered CVMR, increases in brain WMH, brain atrophy, and cognitive decline in older adults. Importantly, one-year moderate-to-vigorous aerobic exercise training increased physical fitness as measured by VO_2peak_, reduced carotid arterial stiffness, decreased CVR, and improved CBF in cognitively normal older adults and patients with MCI [33, 35]. In addition, cognitive performance (memory and executive function) was preserved or improved slightly with exercise training [33, 35]. Furthermore, aerobic exercise improved CVMR during hypo- and hypercapnia, consistent with reductions in the basal cerebral vasomotor tone and CVR [34]. We also observed the presence of a potential “dose-response” relationship between changes in cardiorespiratory fitness and cerebrovascular function. These findings collectively support the hypothesis that aerobic exercise training improves cerebrovascular function in older adults by reducing central arterial stiffness which may benefit cognitive function. Thus, further studies are needed to confirm and expand these observations to better understand the underlying vascular mechanisms of aerobic exercise training in improving cognitive function in older adults and in dementia prevention.

## Conclusions

This review summarized the evidence in support of the hypothesis that increases in central arterial stiffness and arterial pulsation with age are associated with cerebrovascular and cognitive dysfunction in older adults. Importantly, we showed that one-year aerobic exercise training reduced central arterial stiffness and CVR and improved CBF in cognitively normal older adults and in patients with MCI which were associated with improvement in cognitive performance. These findings provide strong evidence that aerobic exercise training improves cerebrovascular function by modifying arterial aging in older adults which may benefit brain health.

## References

[1] Alzheimer’sAssociation (2023). 2023 Alzheimer's disease facts and figures. Alzheimers Dement. 10.1002/alz.13016 36918389

[2] Park DC, Reuter-Lorenz P (2009). The adaptive brain: aging and neurocognitive scaffolding. Annu Rev Psychol, 60:173-196. 19035823 10.1146/annurev.psych.59.103006.093656 PMC3359129

[3] Hoilund-Carlsen PF, Revheim ME, Costa T, Alavi A, Kepp KP, Sensi SL, et al. (2023). Passive Alzheimer's immunotherapy: A promising or uncertain option? Ageing Res Rev, 90:101996. 37414156 10.1016/j.arr.2023.101996

[4] Iadecola C (2004). Neurovascular regulation in the normal brain and in Alzheimer's disease. Nat Rev Neurosci, 5:347-360. 15100718 10.1038/nrn1387

[5] Kivipelto M, Mangialasche F, Ngandu T (2018). Lifestyle interventions to prevent cognitive impairment, dementia and Alzheimer disease. Nat Rev Neurol, 14:653-666. 30291317 10.1038/s41582-018-0070-3

[6] Kisler K, Nelson AR, Montagne A, Zlokovic BV (2017). Cerebral blood flow regulation and neurovascular dysfunction in Alzheimer disease. Nat Rev Neurosci, 18:419-434. 28515434 10.1038/nrn.2017.48 PMC5759779

[7] de la Torre JC (2004). Is Alzheimer's disease a neurodegenerative or a vascular disorder? Data, dogma, and dialectics. Lancet Neurol, 3:184-190. 14980533 10.1016/S1474-4422(04)00683-0

[8] Kapasi A, DeCarli C, Schneider JA (2017). Impact of multiple pathologies on the threshold for clinically overt dementia. Acta Neuropathol, 134:171-186. 28488154 10.1007/s00401-017-1717-7 PMC5663642

[9] Mitchell GF (2008). Effects of central arterial aging on the structure and function of the peripheral vasculature: implications for end-organ damage. Journal of applied physiology, 105:1652-1660. 18772322 10.1152/japplphysiol.90549.2008 PMC2584844

[10] O'Rourke MF, Hashimoto J (2007). Mechanical factors in arterial aging: a clinical perspective. Journal of the American College of Cardiology, 50:1-13. 17601538 10.1016/j.jacc.2006.12.050

[11] Thorin-Trescases N, de Montgolfier O, Pincon A, Raignault A, Caland L, Labbe P, et al. (2018). Impact of pulse pressure on cerebrovascular events leading to age-related cognitive decline. Am J Physiol Heart Circ Physiol, 314:H1214-H1224. 29451817 10.1152/ajpheart.00637.2017 PMC6032083

[12] Badji A, Sabra D, Bherer L, Cohen-Adad J, Girouard H, Gauthier CJ (2019). Arterial stiffness and brain integrity: A review of MRI findings. Ageing Res Rev, 53:100907. 31063866 10.1016/j.arr.2019.05.001

[13] Cooper LL, O'Donnell A, Beiser AS, Thibault EG, Sanchez JS, Benjamin EJ, et al. (2022). Association of Aortic Stiffness and Pressure Pulsatility With Global Amyloid-beta and Regional Tau Burden Among Framingham Heart Study Participants Without Dementia. JAMA Neurol, 79:710-719. 35666520 10.1001/jamaneurol.2022.1261 PMC9171656

[14] Singer J, Trollor JN, Baune BT, Sachdev PS, Smith E (2014). Arterial stiffness, the brain and cognition: a systematic review. Ageing Res Rev, 15:16-27. 24548924 10.1016/j.arr.2014.02.002

[15] van Sloten TT, Protogerou AD, Henry RM, Schram MT, Launer LJ, Stehouwer CD (2015). Association between arterial stiffness, cerebral small vessel disease and cognitive impairment: A systematic review and meta-analysis. Neurosci Biobehav Rev, 53:121-130. 25827412 10.1016/j.neubiorev.2015.03.011 PMC5314721

[16] Beishon L, Haunton VJ, Panerai RB, Robinson TG (2017). Cerebral Hemodynamics in Mild Cognitive Impairment: A Systematic Review. J Alzheimers Dis, 59:369-385. 28671118 10.3233/JAD-170181

[17] de Eulate RG, Goni I, Galiano A, Vidorreta M, Recio M, Riverol M, et al. (2017). Reduced Cerebral Blood Flow in Mild Cognitive Impairment Assessed Using Phase-Contrast MRI. J Alzheimers Dis, 58:585-595. 28453476 10.3233/JAD-161222

[18] Iturria-Medina Y, Sotero RC, Toussaint PJ, Mateos-Perez JM, Evans AC, Alzheimer's Disease Neuroimaging I (2016). Early role of vascular dysregulation on late-onset Alzheimer's disease based on multifactorial data-driven analysis. Nat Commun, 7:11934. 27327500 10.1038/ncomms11934 PMC4919512

[19] Mitchell GF, van Buchem MA, Sigurdsson S, Gotal JD, Jonsdottir MK, Kjartansson O, et al. (2011). Arterial stiffness, pressure and flow pulsatility and brain structure and function: the Age, Gene/Environment Susceptibility--Reykjavik study. Brain : a journal of neurology, 134:3398-3407. 22075523 10.1093/brain/awr253 PMC3212721

[20] Barnes JN, Pearson AG, Corkery AT, Eisenmann NA, Miller KB (2021). Exercise, Arterial Stiffness, and Cerebral Vascular Function: Potential Impact on Brain Health. J Int Neuropsychol Soc, 27:761-775. 33952365 10.1017/S1355617721000394 PMC8496967

[21] Bliss ES, Wong RH, Howe PR, Mills DE (2021). Benefits of exercise training on cerebrovascular and cognitive function in ageing. J Cereb Blood Flow Metab, 41:447-470. 32954902 10.1177/0271678X20957807 PMC7907999

[22] De la Rosa A, Olaso-Gonzalez G, Arc-Chagnaud C, Millan F, Salvador-Pascual A, Garcia-Lucerga C, et al. (2020). Physical exercise in the prevention and treatment of Alzheimer's disease. J Sport Health Sci, 9:394-404. 32780691 10.1016/j.jshs.2020.01.004 PMC7498620

[23] Kleinloog JPD, Nijssen KMR, Mensink RP, Joris PJ (2023). Effects of Physical Exercise Training on Cerebral Blood Flow Measurements: A Systematic Review of Human Intervention Studies. Int J Sport Nutr Exerc Metab, 33:47-59. 36170974 10.1123/ijsnem.2022-0085

[24] Smith EC, Pizzey FK, Askew CD, Mielke GI, Ainslie PN, Coombes JS, et al. (2021). Effects of cardiorespiratory fitness and exercise training on cerebrovascular blood flow and reactivity: a systematic review with meta-analyses. Am J Physiol Heart Circ Physiol, 321:H59-H76. 34018848 10.1152/ajpheart.00880.2020

[25] Tarumi T, Zhang R (2015). The Role of Exercise-Induced Cardiovascular Adaptation in Brain Health. Exerc Sport Sci Rev, 43:181-189. 26196870 10.1249/JES.0000000000000063

[26] Stillman CM, Esteban-Cornejo I, Brown B, Bender CM, Erickson KI (2020). Effects of Exercise on Brain and Cognition Across Age Groups and Health States. Trends Neurosci, 43:533-543. 32409017 10.1016/j.tins.2020.04.010 PMC9068803

[27] Seals DR, Desouza CA, Donato AJ, Tanaka H (2008). Habitual exercise and arterial aging. J Appl Physiol (1985), 105:1323-1332. 18583377 10.1152/japplphysiol.90553.2008 PMC2576026

[28] Shibata S, Fujimoto N, Hastings JL, Carrick-Ranson G, Bhella PS, Hearon CM Jr., et al. (2018). The effect of lifelong exercise frequency on arterial stiffness. J Physiol, 596:2783-2795. 29781119 10.1113/JP275301 PMC6046080

[29] Tanaka H (2019). Antiaging Effects of Aerobic Exercise on Systemic Arteries. Hypertension, 74:237-243. 31256721 10.1161/HYPERTENSIONAHA.119.13179

[30] Tomoto T, Lu M, Khan AM, Liu J, Pasha EP, Tarumi T, et al. (2023). Cerebral blood flow and cerebrovascular resistance across the adult lifespan: A multimodality approach. J Cereb Blood Flow Metab, 43:962-976. 36708213 10.1177/0271678X231153741 PMC10196748

[31] Tomoto T, Riley J, Turner M, Zhang R, Tarumi T (2020). Cerebral vasomotor reactivity during hypo- and hypercapnia across the adult lifespan. J Cereb Blood Flow Metab, 40:600-610. 30764704 10.1177/0271678X19828327 PMC7026853

[32] Tomoto T, Tarumi T, Zhang R (2023). Central arterial stiffness, brain white matter hyperintensity and total brain volume across the adult lifespan. J Hypertens, 41:819-829. 36883450 10.1097/HJH.0000000000003404 PMC10079586

[33] Tomoto T, Liu J, Tseng BY, Pasha EP, Cardim D, Tarumi T, et al. (2021). One-Year Aerobic Exercise Reduced Carotid Arterial Stiffness and Increased Cerebral Blood Flow in Amnestic Mild Cognitive Impairment. J Alzheimers Dis, 80:841-853. 33579857 10.3233/JAD-201456

[34] Tomoto T, Tarumi T, Chen JN, Hynan LS, Cullum CM, Zhang R (2021). One-year aerobic exercise altered cerebral vasomotor reactivity in mild cognitive impairment. J Appl Physiol (1985), 131:119-130. 34013755 10.1152/japplphysiol.00158.2021 PMC8325610

[35] Tomoto T, Verma A, Kostroske K, Tarumi T, Patel NR, Pasha EP, et al. (2023). One-year aerobic exercise increases cerebral blood flow in cognitively normal older adults. J Cereb Blood Flow Metab, 43:404-418. 36250505 10.1177/0271678X221133861 PMC9941859

[36] Chirinos JA, Segers P, Hughes T, Townsend R (2019). Large-Artery Stiffness in Health and Disease: JACC State-of-the-Art Review. J Am Coll Cardiol, 74:1237-1263. 31466622 10.1016/j.jacc.2019.07.012 PMC6719727

[37] Belz GG (1995). Elastic properties and Windkessel function of the human aorta. Cardiovasc Drugs Ther, 9:73-83. 7786838 10.1007/BF00877747

[38] Van Bortel LM, Laurent S, Boutouyrie P, Chowienczyk P, Cruickshank JK, De Backer T, et al. (2012). Expert consensus document on the measurement of aortic stiffness in daily practice using carotid-femoral pulse wave velocity. J Hypertens, 30:445-448. 22278144 10.1097/HJH.0b013e32834fa8b0

[39] Hirai T, Sasayama S, Kawasaki T, Yagi S (1989). Stiffness of systemic arteries in patients with myocardial infarction. A noninvasive method to predict severity of coronary atherosclerosis. Circulation, 80:78-86. 2610739 10.1161/01.cir.80.1.78

[40] Tomoto T, Sugawara J, Tarumi T, Chiles C, Curtis B, Pasha EP, et al. (2020). Carotid Arterial Stiffness and Cerebral Blood Flow in Amnestic Mild Cognitive Impairment. Curr Alzheimer Res, 17:1115-1125. 33441064 10.2174/1567205018666210113155646

[41] Jokinen H, Lipsanen J, Schmidt R, Fazekas F, Gouw AA, van der Flier WM, et al. (2012). Brain atrophy accelerates cognitive decline in cerebral small vessel disease: the LADIS study. Neurology, 78:1785-1792. 22592361 10.1212/WNL.0b013e3182583070

[42] Muller M, Appelman AP, van der Graaf Y, Vincken KL, Mali WP, Geerlings MI (2011). Brain atrophy and cognition: interaction with cerebrovascular pathology? Neurobiol Aging, 32:885-893. 19520460 10.1016/j.neurobiolaging.2009.05.005

[43] Claassen J, Thijssen DHJ, Panerai RB, Faraci FM (2021). Regulation of cerebral blood flow in humans: physiology and clinical implications of autoregulation. Physiol Rev, 101:1487-1559. 33769101 10.1152/physrev.00022.2020 PMC8576366

[44] Fantini S, Sassaroli A, Tgavalekos KT, Kornbluth J (2016). Cerebral blood flow and autoregulation: current measurement techniques and prospects for noninvasive optical methods. Neurophotonics, 3:031411. 27403447 10.1117/1.NPh.3.3.031411 PMC4914489

[45] Liu Y, Zhu X, Feinberg D, Guenther M, Gregori J, Weiner MW, et al. (2012). Arterial spin labeling MRI study of age and gender effects on brain perfusion hemodynamics. Magn Reson Med, 68:912-922. 22139957 10.1002/mrm.23286

[46] Parkes LM, Rashid W, Chard DT, Tofts PS (2004). Normal cerebral perfusion measurements using arterial spin labeling: reproducibility, stability, and age and gender effects. Magn Reson Med, 51:736-743. 15065246 10.1002/mrm.20023

[47] Yew B, Nation DA, Alzheimer's Disease Neuroimaging I (2017). Cerebrovascular resistance: effects on cognitive decline, cortical atrophy, and progression to dementia. Brain, 140:1987-2001. 28575149 10.1093/brain/awx112 PMC6059092

[48] Christie IN, Windsor R, Mutsaerts HJ, Tillin T, Sudre CH, Hughes AD, et al. (2022). Cerebral perfusion in untreated, controlled, and uncontrolled hypertension. J Cereb Blood Flow Metab, 42:2188-2190. 36113055 10.1177/0271678X221124644 PMC7613835

[49] Giannopoulos S, Katsanos AH, Tsivgoulis G, Marshall RS (2012). Statins and cerebral hemodynamics. J Cereb Blood Flow Metab, 32:1973-1976. 22929438 10.1038/jcbfm.2012.122 PMC3494001

[50] van Rijssel AE, Stins BC, Beishon LC, Sanders ML, Quinn TJ, Claassen J, et al. (2022). Effect of Antihypertensive Treatment on Cerebral Blood Flow in Older Adults: a Systematic Review and Meta-Analysis. Hypertension, 79:1067-1078. 35193363 10.1161/HYPERTENSIONAHA.121.18255 PMC8997667

[51] Liu J, Zhu YS, Khan MA, Brunk E, Martin-Cook K, Weiner MF, et al. (2014). Global brain hypoperfusion and oxygenation in amnestic mild cognitive impairment. Alzheimers Dement, 10:162-170. 23871763 10.1016/j.jalz.2013.04.507 PMC3859724

[52] Pasha EP, Rutjes E, Tomoto T, Tarumi T, Stowe A, Claassen J, et al. (2020). Carotid Stiffness is Associated with Brain Amyloid-beta Burden in Amnestic Mild Cognitive Impairment. J Alzheimers Dis, 74:925-935. 32083583 10.3233/JAD-191073

[53] Small GW, Kepe V, Ercoli LM, Siddarth P, Bookheimer SY, Miller KJ, et al. (2006). PET of brain amyloid and tau in mild cognitive impairment. N Engl J Med, 355:2652-2663. 17182990 10.1056/NEJMoa054625

[54] Kety SS, Schmidt CF (1948). The Effects of Altered Arterial Tensions of Carbon Dioxide and Oxygen on Cerebral Blood Flow and Cerebral Oxygen Consumption of Normal Young Men. J Clin Invest, 27:484-492. 16695569 10.1172/JCI101995 PMC439519

[55] Reivich M (1964). Arterial Pco2 and Cerebral Hemodynamics. Am J Physiol, 206:25-35. 14117646 10.1152/ajplegacy.1964.206.1.25

[56] Hosford PS, Wells JA, Nizari S, Christie IN, Theparambil SM, Castro PA, et al. (2022). CO(2) signaling mediates neurovascular coupling in the cerebral cortex. Nat Commun, 13:2125. 35440557 10.1038/s41467-022-29622-9 PMC9019094

[57] Willie CK, Colino FL, Bailey DM, Tzeng YC, Binsted G, Jones LW, et al. (2011). Utility of transcranial Doppler ultrasound for the integrative assessment of cerebrovascular function. J Neurosci Methods, 196:221-237. 21276818 10.1016/j.jneumeth.2011.01.011

[58] Bliss ES, Biki SM, Wong RHX, Howe PRC, Mills DE (2023). The benefits of regular aerobic exercise training on cerebrovascular function and cognition in older adults. Eur J Appl Physiol, 123:1323-1342. 36801969 10.1007/s00421-023-05154-y PMC9938957

[59] Bliss ES, Wong RHX, Howe PRC, Mills DE (2022). The Effects of Aerobic Exercise Training on Cerebrovascular and Cognitive Function in Sedentary, Obese, Older Adults. Front Aging Neurosci, 14:892343. 35663579 10.3389/fnagi.2022.892343 PMC9158462

[60] Phillips AA, Chan FH, Zheng MM, Krassioukov AV, Ainslie PN (2016). Neurovascular coupling in humans: Physiology, methodological advances and clinical implications. J Cereb Blood Flow Metab, 36:647-664. 26661243 10.1177/0271678X15617954 PMC4821024

[61] Wong RHX, Evans HM, Howe PRC (2016). Poor cerebrovascular function is an early marker of cognitive decline in healthy postmenopausal women. Alzheimers Dement (N Y), 2:162-168. 29067303 10.1016/j.trci.2016.07.003 PMC5651351

[62] Claassen JA, Zhang R, Fu Q, Witkowski S, Levine BD (2007). Transcranial Doppler estimation of cerebral blood flow and cerebrovascular conductance during modified rebreathing. J Appl Physiol (1985), 102:870-877. 17110510 10.1152/japplphysiol.00906.2006

[63] Brothers RM, Lucas RA, Zhu YS, Crandall CG, Zhang R (2014). Cerebral vasomotor reactivity: steady-state versus transient changes in carbon dioxide tension. Exp Physiol, 99:1499-1510. 25172891 10.1113/expphysiol.2014.081190 PMC4218865

[64] Gotoh F, Meyer JS, Takagi Y (1965). Cerebral Effects of Hyperventilation in Man. Arch Neurol, 12:410-423. 14264873 10.1001/archneur.1965.00460280080008

[65] Yamaguchi F, Meyer JS, Sakai F, Yamamoto M (1979). Normal human aging and cerebral vasoconstrictive responses to hypocapnia. J Neurol Sci, 44:87-94. 512693 10.1016/0022-510x(79)90226-0

[66] Cullen DJ, Eger EI 2nd (1974). Cardiovascular effects of carbon dioxide in man. Anesthesiology, 41:345-349. 4412334 10.1097/00000542-197410000-00006

[67] Provinciali L, Minciotti P, Ceravolo G, Angeleri F, Sanguinetti CM (1990). Transcranial Doppler sonography as a diagnostic tool in vascular dementia. Eur Neurol, 30:98-103. 2187699 10.1159/000117320

[68] Anzola GP, Galluzzi S, Mazzucco S, Frisoni GB (2011). Autonomic dysfunction in mild cognitive impairment: a transcranial Doppler study. Acta Neurol Scand, 124:403-409. 22017634 10.1111/j.1600-0404.2011.01495.x

[69] Galvin SD, Celi LA, Thomas KN, Clendon TR, Galvin IF, Bunton RW, et al. (2010). Effects of age and coronary artery disease on cerebrovascular reactivity to carbon dioxide in humans. Anaesth Intensive Care, 38:710-717. 20715736 10.1177/0310057X1003800415

[70] Ito H, Kanno I, Ibaraki M, Hatazawa J (2002). Effect of aging on cerebral vascular response to Paco2 changes in humans as measured by positron emission tomography. J Cereb Blood Flow Metab, 22:997-1003. 12172385 10.1097/00004647-200208000-00011

[71] Zhu YS, Tarumi T, Tseng BY, Palmer DM, Levine BD, Zhang R (2013). Cerebral vasomotor reactivity during hypo- and hypercapnia in sedentary elderly and Masters athletes. J Cereb Blood Flow Metab, 33:1190-1196. 23591649 10.1038/jcbfm.2013.66 PMC3734768

[72] Tomoto T, Tarumi T, Chen J, Pasha EP, Cullum CM, Zhang R (2020). Cerebral Vasomotor Reactivity in Amnestic Mild Cognitive Impairment. J Alzheimers Dis, 77:191-202. 32716360 10.3233/JAD-200194

[73] Guadagni V, Drogos LL, Tyndall AV, Davenport MH, Anderson TJ, Eskes GA, et al. (2020). Aerobic exercise improves cognition and cerebrovascular regulation in older adults. Neurology, 94:e2245-e2257. 32404355 10.1212/WNL.0000000000009478 PMC7357295

[74] Tarumi T, Patel NR, Tomoto T, Pasha E, Khan AM, Kostroske K, et al. (2022). Aerobic exercise training and neurocognitive function in cognitively normal older adults: A one-year randomized controlled trial. J Intern Med, 292:788-803. 35713933 10.1111/joim.13534 PMC9588521

[75] Morland C, Andersson KA, Haugen OP, Hadzic A, Kleppa L, Gille A, et al. (2017). Exercise induces cerebral VEGF and angiogenesis via the lactate receptor HCAR1. Nat Commun, 8:15557. 28534495 10.1038/ncomms15557 PMC5457513

[76] Jezzard P, Chappell MA, Okell TW (2018). Arterial spin labeling for the measurement of cerebral perfusion and angiography. J Cereb Blood Flow Metab, 38:603-626. 29168667 10.1177/0271678X17743240 PMC5888859

[77] Ivey FM, Ryan AS, Hafer-Macko CE, Macko RF (2011). Improved cerebral vasomotor reactivity after exercise training in hemiparetic stroke survivors. Stroke, 42:1994-2000. 21636819 10.1161/STROKEAHA.110.607879

[78] Murrell CJ, Cotter JD, Thomas KN, Lucas SJ, Williams MJ, Ainslie PN (2013). Cerebral blood flow and cerebrovascular reactivity at rest and during sub-maximal exercise: effect of age and 12-week exercise training. Age, 35:905-920. 22669592 10.1007/s11357-012-9414-x PMC3636405

[79] Erickson KI, Voss MW, Prakash RS, Basak C, Szabo A, Chaddock L, et al. (2011). Exercise training increases size of hippocampus and improves memory. Proc Natl Acad Sci U S A, 108:3017-3022. 21282661 10.1073/pnas.1015950108 PMC3041121

[80] Lee IM (2007). Dose-response relation between physical activity and fitness: even a little is good; more is better. JAMA, 297:2137-2139. 17507351 10.1001/jama.297.19.2137

[81] Laurin D, Verreault R, Lindsay J, MacPherson K, Rockwood K (2001). Physical activity and risk of cognitive impairment and dementia in elderly persons. Arch Neurol, 58:498-504. 11255456 10.1001/archneur.58.3.498

[82] Wendell CR, Gunstad J, Waldstein SR, Wright JG, Ferrucci L, Zonderman AB (2014). Cardiorespiratory fitness and accelerated cognitive decline with aging. J Gerontol A Biol Sci Med Sci, 69:455-462. 24192540 10.1093/gerona/glt144 PMC3968827

[83] Vidoni ED, Johnson DK, Morris JK, Van Sciver A, Greer CS, Billinger SA, et al. (2015). Dose-Response of Aerobic Exercise on Cognition: A Community-Based, Pilot Randomized Controlled Trial. PLoS One, 10:e0131647. 26158265 10.1371/journal.pone.0131647 PMC4497726

[84] Tseng BY, Uh J, Rossetti HC, Cullum CM, Diaz-Arrastia RF, Levine BD, et al. (2013). Masters athletes exhibit larger regional brain volume and better cognitive performance than sedentary older adults. J Magn Reson Imaging, 38:1169-1176. 23908143 10.1002/jmri.24085 PMC3812419

[85] Tseng BY, Gundapuneedi T, Khan MA, Diaz-Arrastia R, Levine BD, Lu H, et al. (2013). White matter integrity in physically fit older adults. Neuroimage, 82:510-516. 23769914 10.1016/j.neuroimage.2013.06.011 PMC3759589

[86] Calverley TA, Ogoh S, Marley CJ, Steggall M, Marchi N, Brassard P, et al. (2020). HIITing the brain with exercise: mechanisms, consequences and practical recommendations. J Physiol, 598:2513-2530. 32347544 10.1113/JP275021

[87] Chapman SB, Aslan S, Spence JS, Defina LF, Keebler MW, Didehbani N, et al. (2013). Shorter term aerobic exercise improves brain, cognition, and cardiovascular fitness in aging. Front Aging Neurosci, 5:75. 24282403 10.3389/fnagi.2013.00075 PMC3825180

